# Combined Internet-Based Cognitive Behavioral Therapy and Face-to-Face Physiotherapy in Primary Health Care for Chronic Widespread Pain: Randomized Controlled Trial

**DOI:** 10.2196/86792

**Published:** 2026-06-29

**Authors:** Anna Bergenheim, Maria EH Larsson, Chan-Mei Ho-Henriksson, Anna Grimby-Ekman, Anna Caroline Larsson, Marie Persson, Sandra Weineland, Lena Nordeman

**Affiliations:** 1 Department of Health and Rehabilitation Sahlgrenska Academy, Institute of Neuroscience and Physiology University of Gothenburg Gothenburg Sweden; 2 Research, Education, Development and Innovation, Primary Health Care Region Västra Götaland Sweden; 3 Närhälsan (Public Healthcare Provider) Region Västra Götaland Sweden; 4 School of Public Health and Community Medicine Sahlgrenska Academy, Institute of Medicine University of Gothenburg Gothenburg Sweden; 5 Department of Psychology University of Gothenburg Gothenburg Sweden

**Keywords:** chronic pain, cognitive behavioral therapy, exercise, internet, physiotherapy

## Abstract

**Background:**

Finding successful treatments for chronic widespread pain (CWP) in primary care is challenging. Interventions addressing both stress and pain may yield synergistic effects. Internet-based cognitive behavioral therapy (iCBT) reduces stress-related pain responses, while physical activity enhances function and resilience. Combined, they may target core CWP mechanisms.

**Objective:**

This study aimed to evaluate the effectiveness of therapist-guided iCBT for stress management combined with physiotherapist-guided physical activity, compared to stand-alone physical activity, on pain and associated symptoms in individuals with CWP.

**Methods:**

Participants with CWP, aged 18-70 years, were recruited in this parallel, multicenter randomized controlled trial via social media in Sweden. The intervention group received a 14-module iCBT program plus a physical activity plan; the control group received the physical activity plan only. Primary outcomes were pain intensity and pain locations. Secondary outcomes included stress, fatigue, depression, and quality of life. Variables were self-assessed using paper questionnaires at baseline and 6 months. Allocation was concealed; participants and researchers were not blinded.

**Results:**

Of 129 participants (64 intervention, 65 control), 82 (64%) completed the 6-month follow-up. In the intervention group, 37% (16/43) completed all 14 iCBT modules. No between-group differences were observed for change in any outcome: pain intensity (mean 1.7, 95% CI –7.5 to 11.0), pain locations (mean –0.8, 95% CI –2.2 to 0.6), Stress and Crisis Inventory (mean 2.0, 95% CI –3.3 to 7.4), Fibromyalgia Impact Questionnaire (mean 2.7, 95% CI –3.7 to 9.2), global fatigue (mean 3.0, 95% CI –7.2 to 13.3), Multidimensional Fatigue Inventory (general fatigue: mean 0.1, 95% CI –1.1 to 1.3; physical: mean 0.4, 95% CI –1.1 to 1.9; mental: mean 0.4, 95% CI –1.1 to 2.0; reduced activity: mean –2.8, 95% CI –0.5 to 2.1; reduced motivation mean –0.5, 95% CI –2.2 to 1.2), Hospital Anxiety and Depression Scale (anxiety: mean –0.8, 95% CI –2.3 to 0.7; depression: mean –0.2, 95% CI –1.7 to 1.3), and Short-Form 36 subscales (physical function: mean 2.9, 95% CI –3.7 to 9.5; role physical: mean –13.1, 95% CI –28.3 to 2.0; role emotional: mean –4.0, 95% CI –22.8 to 14.8; energy/fatigue: mean 3.4, 95% CI –4.0 to 10.8; emotional well-being: mean –0.5, 95% CI –8.2 to 7.3; social functioning: mean –4.6, 95% CI –15.0 to 5.8; pain: mean –3.1, 95% CI –9.8 to 3.6; general health: mean –2.2, 95% CI –9.2 to 4.9). Both groups improved across several outcomes. Main goals were attained by 37% (17/46) in the intervention group vs 19% (8/42) in controls (*P*=.02), and intermediate goals by 54% (25/46) vs 36% (15/42; *P*=.01).

**Conclusions:**

This study novelly examines stress-targeting interventions for pain. Clinicians could focus on tailored physical activity plans while considering optional stress management to support behavioral change and goal attainment. Due to high loss to follow-up, results should be interpreted cautiously.

**Trial Registration:**

ClinicalTrials.gov NCT04624139; https://clinicaltrials.gov/study/NCT04624139

## Introduction

Chronic widespread pain (CWP) is pain that persists for more than 3 months, occurring above and below the waist, affecting both the right and left sides of the body, and involving axial pain [1]. The prevalence of CWP is estimated to be between 8% and 11% in the western world, being about twice as common among women as men [2]. Most patients with CWP are managed in primary health care [3], and in addition to negatively impacting the individual’s quality of life, CWP imposes a significant economic burden on society [4]. CWP is associated with stiffness, severe fatigue, cognitive difficulties, and psychological distress [5]. The etiology of CWP is not fully understood, but previous research describes various physiological abnormalities, including central sensitization and impaired pain inhibition [6]. Additionally, heightened pain sensitivity in chronic pain populations is linked to dysregulation of the autonomic nervous system and the hypothalamic-pituitary-adrenal axis, although evidence is limited [7]. In women with fibromyalgia, acute stress has been found to be a contributor to momentary pain, while acute pain did not predict momentary stress in the same way [8]. Several demographic, psychosocial, and health-related factors, including female sex, age, family history of pain, depressed mood, widespread pain distribution, poor sleep, obesity, and chronic disease, have been suggested to increase the risk of both progression to and persistence of CWP [9,10]. Additionally, CWP is linked to low physical activity, which has been found to be associated with higher levels of perceived stress [11]. A previous study, examining the long-term symptom changes and predictors of improvement in women with CWP, indicated that a lower degree of stress symptoms at baseline predicted a substantial reduction in pain intensity after 12 years [12]. This finding further strengthens the evidence for an association between stress and pain and highlights the importance of underlying stressors that may trigger, perpetuate, or exacerbate pain.

Fibromyalgia constitutes a subgroup of CWP with more severe symptoms, and although there are criteria for diagnosis, there is not a distinct line for when CWP could be considered as fibromyalgia, since they both lie within the spectrum of nociplastic pain [13], and similar treatment strategies can be applied. European guidelines for treating CWP and fibromyalgia recommend nonpharmacological treatments primarily, such as physical exercise and cognitive behavioral therapy (CBT) [14]. Physical activity contributes to positive outcomes for both stress-related illnesses and chronic pain [15,16]. Various forms of physical exercise show positive effects on symptoms and overall health in individuals with CWP and fibromyalgia [15], and evidence suggests that physical activity could have a positive influence on the autonomic nervous system [17]. Consideration of patients’ personal preferences and available resources may help guide the selection of appropriate exercise interventions. Therapist-guided internet-based CBT (iCBT) has demonstrated positive effects for patients with chronic pain [18] and with stress-related illness [19,20]. Combined, CBT and physical exercise have been found to yield small to moderate improvements in pain intensity, functional disability, mental health, and quality of life in patients with chronic pain compared with active control treatments [21]. However, CBT interventions in previous studies have been heterogeneous in format, content, and provider type [21]. Moreover, despite growing evidence linking stress to pain modulation in CWP [7], few interventions have explicitly targeted stress reduction as a primary mechanism. Likewise, although physical activity is recommended, little is known about the added value of combining structured physical activity with stress-focused iCBT in individuals with CWP.

ICBT is an accessible form of CBT that has demonstrated effects comparable to those of face-to-face CBT in populations with primarily mental health disorders [22]. Stress reduction through iCBT could enhance pain modulation by reducing physiological stress responses, while person-centered physical activity can further improve physical function and resilience against stress. The combination of these 2 evidence-based strategies is therefore of interest, as it may address both the psychological and physiological mechanisms that maintain CWP. The novelty of this project is that the iCBT intervention targets stress management, and not primarily pain management.

This study aimed to evaluate the effectiveness of a therapist-guided iCBT program for stress management, combined with a face-to-face physiotherapist-guided physical activity plan, compared to a control group receiving the physical activity plan as a stand-alone treatment. It was hypothesized that the combined intervention would lead to greater improvements in pain, stress-related symptoms, psychological distress, and physical activity in individuals with CWP.

## Methods

### Trial Design

#### Overview

This was a multicenter randomized controlled trial (RCT) with parallel groups, conducted at 4 sites in southwestern Sweden and online, stratified by sex and total score on the Stress and Crisis Inventory (SCI)-93; <50 or ≥50, allocation ratio 1:1. The study is reported in accordance with the Consolidated Standards of Reporting Trials (CONSORT) 2025 guidelines for reporting RCTs [23] (Multimedia Appendix 1), and the Consolidated Standards of Reporting Trials of Electronic and Mobile Health Applications and Online Telehealth (CONSORT-EHEALTH) guidelines [24] (Multimedia Appendix 2).

#### Changes to Trial Protocol

The actual study start date for participant enrollment was November 4, 2020, which also corresponded to the originally estimated start date. However, this date was not formally updated from “Anticipated” to “Actual” in the trial registry until April 26, 2023, due to an administrative oversight. Recruitment was stopped after the inclusion of 129 participants. Recruitment was substantially affected by the COVID-19 pandemic, which slowed the inclusion process and prolonged the recruitment period. After approximately 2 years of recruitment, it was decided to stop further inclusion to ensure completion of the study within the available funding period and project timeline. This study focuses explicitly on the baseline and 6-month follow-up data. The 12-month follow-up data were not included in the primary analyses because the attrition rate at 12 months was substantial (66/129, 51%), which was considered to limit the validity and interpretability of analyses of intervention effects. The 12-month data are instead reserved for separate, exploratory longitudinal trajectory substudies. Due to the high attrition observed at the 12-month mark, the planned 24-month follow-up was deemed statistically unfeasible and was not conducted; the trial was formally concluded after the completion of the 12-month follow-up assessments. The Short Form-36 (SF-36) version 1 [25] was used instead of Research and Development-36 [26] due to a miscommunication during study implementation. As the questions in the SF-36 version 1 are identical to the Research and Development-36 in content and assess the same domains of health-related quality of life, this change was not considered to affect the study outcomes. The Fibromyalgia Impact Questionnaire (FIQ) [27,28] total score was added after publication of the study protocol to provide a broader measure of overall disease impact and health status, thereby enabling a more comprehensive assessment of the burden of CWP. Additional analyses not mentioned in the study protocol were conducted to investigate differences between groups in participant goal attainment and achievement of the World Health Organization (WHO) recommendations for physical activity [29].

#### Trial Setting

The physiotherapy intervention was conducted face-to-face at primary care rehabilitation centers in 3 cities in western Sweden. The iCBT was delivered online.

#### Patient and Public Involvement

There was no patient or public involvement in the design, conduct, or reporting of the trial.

### Eligibility Criteria

Women and men aged 18-70 years who met the American College of Rheumatology (ACR) 1990 criteria for CWP—pain persisting for more than 3 months, occurring in areas both above and below the waist, on both the right and left sides of the body, and including axial pain [1]—were included in the study. In Sweden, fibromyalgia is clinically diagnosed according to the ACR 1990 criteria.

Participants were excluded for the following reasons: serious physical or psychological disease or other conditions with restrictions on physical activity, not having a smartphone or computer, ongoing psychotherapy or physiotherapy, and insufficient knowledge of the Swedish language for completing the questionnaires and participating in the iCBT. Participants who had a score of >14 on the subscale for anxiety or depression in the Hospital Anxiety and Depression Scale (HADS) were contacted before randomization by a psychologist by telephone for an assessment about the severity of their psychological distress, and if needed, they could be referred to a relevant service within primary health care.

### Recruitment

People with CWP were recruited via social media advertisements on Facebook and Instagram in western Sweden between November 2020 and October 2022. They responded to the advertisement on social media by sending an email to the research project inbox. Research assistant 1 sent written study information via email. Individuals who expressed interest after reading the information were screened for eligibility by research assistant 1 during a telephone call. If the person was eligible, the written consent form, along with a battery of paper-based baseline questionnaires, was sent home by mail to the participant, and the participant returned the forms by mail.

### Intervention

#### Overview

**Figure 1:**
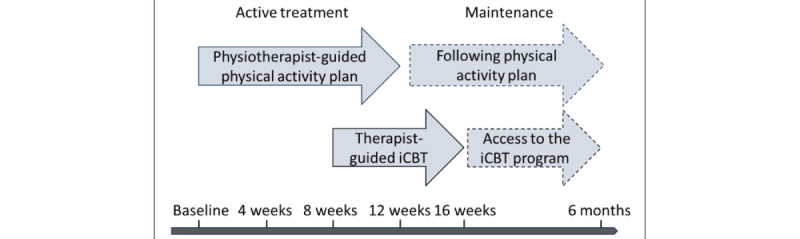
Illustration of the starting points and duration of internet-based cognitive behavioral therapy (iCBT) and face-to-face physical activity plan, delivered in parallel in the combined intervention. A randomized controlled trial in Swedish primary health care, including participants with chronic widespread pain.

The description of the intervention follows the TIDieR (Template for Intervention Description and Replication) checklist [30] (Multimedia Appendix 3). The combined intervention of therapist-guided iCBT for stress and face-to-face physiotherapist-guided physical activity plan was designed to affect both psychological and physiological aspects of stress and pain. The intervention is grounded in proposed biopsychosocial mechanisms of action, suggesting that CWP is partly maintained by stress-related physiological dysregulation and maladaptive behavioral patterns [31]. Accordingly, the combination of stress-focused iCBT and structured physical activity targets key biological, psychological, and behavioral mechanisms underlying pain. The iCBT was delivered online and guided by a psychologist. The physiotherapy was conducted face-to-face at 4 rehabilitation clinics in primary health care in Region Västra Götaland in western Sweden. The iCBT and the physiotherapy were delivered in parallel with different starting points and were not integrated. The starting point was at baseline for the physical activity plan and at 8 weeks for the iCBT program. The starting points could differ by up to 2 weeks. The time points for the intervention components are illustrated in Figure 1. No modifications to the interventions were made during the study period. Participation in the intervention and access to the iCBT program were free of charge, and the participants received no compensation.

#### Therapist-Guided iCBT Designed for Stress-Related Illness

The iCBT program used in this study is a stress management program. A previous version of the program has been used and was well accepted among patients with stress-related disorders, resulting in improvements in perceived stress, anxiety, and depression, although it was not superior to a waitlist control group [32]. The program has since been updated, but no version of the stress management program has previously been evaluated in patients whose primary condition is chronic pain. The iCBT started about 8 weeks after baseline. The 8-week digital program was based on traditional CBT techniques, driven by experiential learning and with a focus on increasing psychological flexibility related to feelings. The iCBT program was developed and provided by Livanda AB. The program consisted of 14 modules addressing the following core processes: behavioral activation and exposure, cognitive restructuring, physiological regulation, mindfulness, acceptance, skills training and problem-solving, as well as maintenance and relapse prevention. Together, these aimed to increase psychological flexibility, reduce avoidance, and improve stress management. The contents of the modules are described in Multimedia Appendix 4. The participants were encouraged to complete 2 modules per week. The program guided participants through understanding stress, setting personal goals, practicing relaxation, and developing long-term coping strategies. Each module is built on the previous one, providing practical tools and exercises to reduce stress and support lasting behavioral change. Therapist support was delivered throughout the iCBT program intervention by 2 psychologists. The psychologists were licensed, trained in CBT, and had completed a 1.5-day training in iCBT before starting the study. The iCBT started with a telephone call with the psychologist. During the call, the iCBT treatment was introduced, explained, and motivated for the participant. The psychologists tracked progress, addressed patient queries, and reached out if engagement declined. Brief telephone check-ins were also conducted at mid- and posttreatment. If the participant followed the time plan for the program, it was expected to be completed after 8 weeks. However, they had access to the program for 6 months and could access the platform at any time and proceed at their own pace. The program included written texts, exercise videos, and audio files.

#### Face-to-Face Physiotherapist-Guided Physical Activity Plan

Participants attended 3 individual 30-minute face-to-face visits with a physiotherapist at baseline, 4 weeks, and 12 weeks, during which a physical activity plan was created, reviewed, and progressively adjusted. The first visit was conducted face-to-face within 2 weeks after baseline. In visit 1 (baseline), the participant and the physiotherapist cocreated an individualized plan of physical activity, according to person-centered principles [33], to be performed at home. The physical activity plan has been used and evaluated in a previous RCT in patients with CWP [34-36]. In this study, the physical activity plan aimed to enable participants to meet the WHO recommendations for weekly physical activity associated with optimal health outcomes: 150-300 minutes of moderate intensity or 75-150 minutes of vigorous intensity physical activity per week, in combination with regular strengthening exercises [29]. The individualized physical activity plan comprised at least 2 components: aerobic exercise and resistance exercise. Aerobic exercise of moderate intensity, aiming toward a total amount of time of 150 minutes a week on moderate intensity or 75 minutes a week on vigorous intensity (or a combination of the two). The resistance exercise program was individualized and performed 2 times a week at a minimum. The exercises started on an individual level with increasing duration and intensity successively over 12 weeks. The physical activity plan also included individual goals that participants aimed to achieve, comprising both main goals and intermediate goals. The main goals referred to overall, long-term outcomes the participant sought through rehabilitation—such as returning to work, improving daily functioning, performing specific tasks important to them, or reducing pain. Intermediate goals were shorter-term, step-by-step objectives that supported progress toward the main goals. These might include improving physical strength, learning pain management techniques, or increasing activity levels. Visit 2 (4 weeks) included follow-up, revision, and progression of the physical activity plan. Visit 3 (12 weeks) included follow-up of attainment of the individual goals and follow-up, revision, and progression of the physical activity plan. The participants were encouraged to continue to follow their plan autonomously.

A total of 9 physiotherapists at the 4 study sites delivered the physiotherapy intervention. All physiotherapists had experience working with chronic pain and stress-related disorders and cocreating physical activity plans with patients in primary health care. The physiotherapists received both oral and written standardized instructions from the principal investigator, outlining the content of each visit and the procedures for documentation.

### Control Group

The control group received the same physiotherapy intervention as the intervention group, but as a stand-alone treatment. It consisted of 3 individual visits: at baseline, 4 weeks, and 12 weeks, during which a physical activity plan was created, revised, and progressively adjusted.

### Fidelity and Adherence to the Intervention

Fidelity was ensured by delivering the iCBT content identically to all participants through the digital platform, while the physical activity plan was implemented according to a standardized template. Adherence to the core components of the intervention was assessed based on the number of completed modules and attendance at all 3 physiotherapist visits.

### Data Collection

The participants completed a battery of paper-based questionnaires at home at baseline and at 6-month follow-up. Follow-up at 6 months was chosen since the participants had access to the iCBT program for 6 months. The questionnaires were sent by mail, with reminders sent by email. The participants completed the same battery of questionnaires after 6 months. They received and returned the questionnaires by mail.

### Primary Outcomes

#### Pain Intensity

Pain intensity assessed with a visual analog scale (VAS; 0-100 mm) included as a subscale in the FIQ. The participants rated their pain intensity during the previous week [28]. The FIQ is a brief, self-administered instrument designed to evaluate the current health status of women with fibromyalgia syndrome. It consists of 10 items that assess various aspects of health and daily functioning, including physical functioning, work status, depression, anxiety, sleep, pain, stiffness, fatigue, and overall well-being. The FIQ was developed and validated for patients with fibromyalgia and CWP [27].

#### Number of Pain Locations (0-18)

The participants marked their painful areas on a pain drawing [37] with 18 predefined body areas.

### Secondary Outcomes

#### Symptoms of Stress (0-140)

SCI-93 consists of 35 items assessing clinical manifestations of stress ranging from 0 (not at all) to 4 (very much). The questions include both physical and mental experiences. The total score ranges from 0 to 140, and higher scores indicate a higher level of stress [38]. The following cutoff values have been recommended [39]: (1) 0-25 points equal a normal stress reaction, (2) 26-50 points indicate a mild stress reaction, (3) 51-75 points represent a moderate stress reaction, (4) 75-100 points demonstrate a severe stress reaction, (5) 101-140 points suggest a very severe stress reaction. The SCI-93 has shown satisfactory known-group validity and test-retest reliability in women with fibromyalgia and CWP [38].

#### Physical Activity (Hours)

Level of physical activity was measured with the Leisure Time Physical Activity Instrument, a questionnaire assessing the amount of physical activity during a typical week. The total score is the number of hours of physical activity [40]. The Leisure Time Physical Activity Instrument has shown satisfactory construct validity and test-retest reliability in women with fibromyalgia [40].

#### Overall Health Status

The FIQ total score provides a comprehensive overview of the impact of fibromyalgia on a patient’s life and is widely used in both clinical practice and research. The total score ranges from 0 to 100, and a higher score indicates more severe symptoms and impairment [27].

#### Fatigue

The Multidimensional Fatigue Inventory (MFI)-20 consists of 20 statements rated on a 5-point Likert scale, assessing various aspects of fatigue experienced over recent days. It produces 5 subscale scores: general fatigue, physical fatigue, mental fatigue, reduced motivation, and reduced activity. Each subscale score ranges from 4 to 20, with higher scores indicating greater levels of fatigue [41]. The MFI-20 has demonstrated satisfactory convergent construct validity and test-retest reliability in individuals with chronic pain conditions [42]. The FIQ subscale for fatigue (VAS 0-100 mm) was also used as a global measure of fatigue during the last week [28].

#### Health-Related Quality of Life

Health-related quality of life was assessed with the SF-36 version 1, a generic instrument that consists of 8 subscales ranging from 0 to 100. A higher score indicates better health-related quality of life [25].

#### Anxiety and Depression

Anxiety and depression were assessed with the HADS, which contains 2 subscales for symptoms of anxiety and depression, ranging from 0 to 21; a higher value means a higher degree of anxiety or depression [43]. A cutoff value of 8 has been suggested to indicate possible anxiety or depression, and a score of ≥15 indicates severe anxiety or depression [44].

#### Participant’s Impression of Change

The participant’s impression of change was assessed at the 6*-*month follow-up with the questionnaire Patient Global Impression of Change (PGIC). The PGIC consists of a single item stating “Since the beginning of the study, my symptoms overall have....” Participants rate this on a 6-point scale ranging from 1 (very much improved) to 6 (very much worsened) [45].

### Additional Outcomes

The additional outcomes were collected during the 12-week visit with the physiotherapist and thus not included as outcomes at the 6*-*month follow-up in the study protocol at ClinicalTrials.gov.

#### Individual Goals in the Physical Activity Plan

At visit 1 with the physiotherapist, the participant set individual goals, both intermediate goals and main goals that the participant wished to achieve. At the 12-week visit, the participants rated whether they had reached their individual goals completely, partially, or not at all.

#### WHO Recommendations for Physical Activity

The physiotherapist assessed during the third visit whether the participant met the WHO recommendations for physical activity [29]. Based on standardized questions, the physiotherapist recorded whether the participant met (1) the guideline of performing strength training for the major muscle groups at least twice per week, and (2) the guideline of performing aerobic physical activity for at least 150 minutes per week at moderate intensity, or alternatively 75 minutes per week at vigorous intensity. Each item was rated by the physiotherapist as “Not at all,” “Partly,” or “Completely” in relation to the guideline.

#### Descriptive Data

Descriptive data were also collected, such as duration of CWP, marital status, and country of birth. Work and sick leave are presented as the current percentage of full-time work (full-time=40 hours of work per week) divided into 4 groups: 0%, 1%-49%, 50%-79%, and 80%-100%.

#### Harms

Information about possible adverse or negative effects was not collected systematically. Physical activity often leads to an initial increase in pain intensity for patients with CWP, and adjusting the intensity of activities is a routine part of the follow-up in the physical activity plan.

### Sample Size

The power calculation was based on the primary outcome variable, pain intensity, which was assessed using a VAS (0-100 mm) [28]. Many factors impact what could be considered a clinically relevant difference in pain intensity between groups, and a suggestion is 20 mm on VAS (16) and a SD of 40-45, which is applied in the power calculation in this study. Statistical significance is *P*=.05 and power=80%. To be able to make analyses of subgroups based on the SCI-93 score, the number of patients was calculated to be 136. To be able to use nonparametric statistics, and to adjust for possible confounders as well as consider 20% dropout at the follow-up, the final sample size was calculated to be 200 participants.

### Randomization

The randomization was carried out by research assistant 2 and performed with block randomization with blocks of 4, with sealed, opaque, sequentially numbered envelopes prepared by a statistician. The randomization was stratified equally by sex and total score on the questionnaire SCI-93 (total score <50 or ≥50). The stratification by level of stress symptoms was conducted to create groups that were as comparable as possible with respect to stress symptom severity, as measured by the SCI-93. This was done in order to enable subgroup analyses of whether the effect of the intervention differed depending on the level of stress symptoms. Neither the research team nor any of the research assistants had access to the random allocation sequence. Participants and researchers were not blinded in this study. The physiotherapists who carried out the intervention in both groups did not know which group the participants were allocated to, if the participant did not mention it themselves.

### Statistical Analyses

The statistical program SPSS (version 28.0.1.1; IBM Corp) was used for data calculations. Descriptive data are presented as mean, median, SD, and range for continuous data and as number and percent for categorical data. The participants were analyzed in trial groups in a randomized trial. Comparisons between groups of change in outcomes were conducted using an independent *t* test. Within-group changes were calculated using a paired *t* test. The results for pain intensity are presented both on group level as change in pain intensity over time between the groups and on an individual level, divided into the proportion of participants in each group achieving moderate (≥30%) and major (≥50%) change in pain intensity [46], with differences between groups calculated using the Pearson chi-square test. Effect sizes representing between-group differences in change from baseline to 6-month follow-up were calculated using Cohen *d* [47]. The primary outcomes, pain intensity and pain locations, were analyzed using linear mixed effects models with repeated measures over time (baseline and 6-month follow-up). Fixed effects included group (intervention vs control), time, and the group-by-time interaction. A random intercept for participants was included to account for within-subject correlation. Potential confounders (sex, age, and pain duration) were first examined by adding each variable separately to model 1. Variables that were significantly associated with pain intensity/pain locations (*P*<.05) were subsequently included in the adjusted model. The final model (Model 2) included group, time, the group-by-time interaction, and significant confounders. Model assumptions were checked by inspection of residuals. Results are presented as *P* values, and statistical significance was set at *P*<.05. Additional analyses of between-group differences at the 12-week visit were conducted for achievement of individual goals using the Mann-Whitney *U* test, and adherence to WHO recommendations for physical activity and muscle-strengthening exercise was assessed using the Pearson chi-square test. All tests were 2-sided, and statistical significance was set at *P*<.05. For the subscales in the MFI-20 and SF-36, missing values were handled by replacing them with the mean of the completed items within that subscale, provided that at least half of the items in the subscale had been completed. This procedure allowed a score for the subscale to be calculated. For the SCI-93, the same procedure was applied; up to 10% missing values were allowed to be replaced with the mean of the completed items. For the FIQ total score, the total was calculated as the mean of the subscale scores. If any subscale score was missing, the mean of the remaining subscales was used. A maximum of 3 out of 10 subscales were allowed to be missing to compute the total FIQ score. Missing data were evaluated using the Little missing completely at random (MCAR) test, including baseline and 6-month follow-up outcome measures, treatment group, and selected demographic variables (age, sex, education, and duration of pain). No interim analyses and stopping guidelines were applied in this study.

### Ethical Considerations

The study was approved by the Swedish ethical review authority (registration number 2023-04031-0). Written informed consent was obtained from all participants on paper. The original informed consent covered the additional analyses, and no further consent was required. Participant privacy and confidentiality were ensured throughout the study. Data were deidentified prior to analysis, and all records were stored securely with access limited to authorized researchers. The study complied with relevant data protection regulations. The manuscript and supplementary materials do not contain any images in which individual participants can be identified. No financial or nonfinancial compensation was provided to participants for their participation in this study.

## Results

### Overview

**Figure 2:**
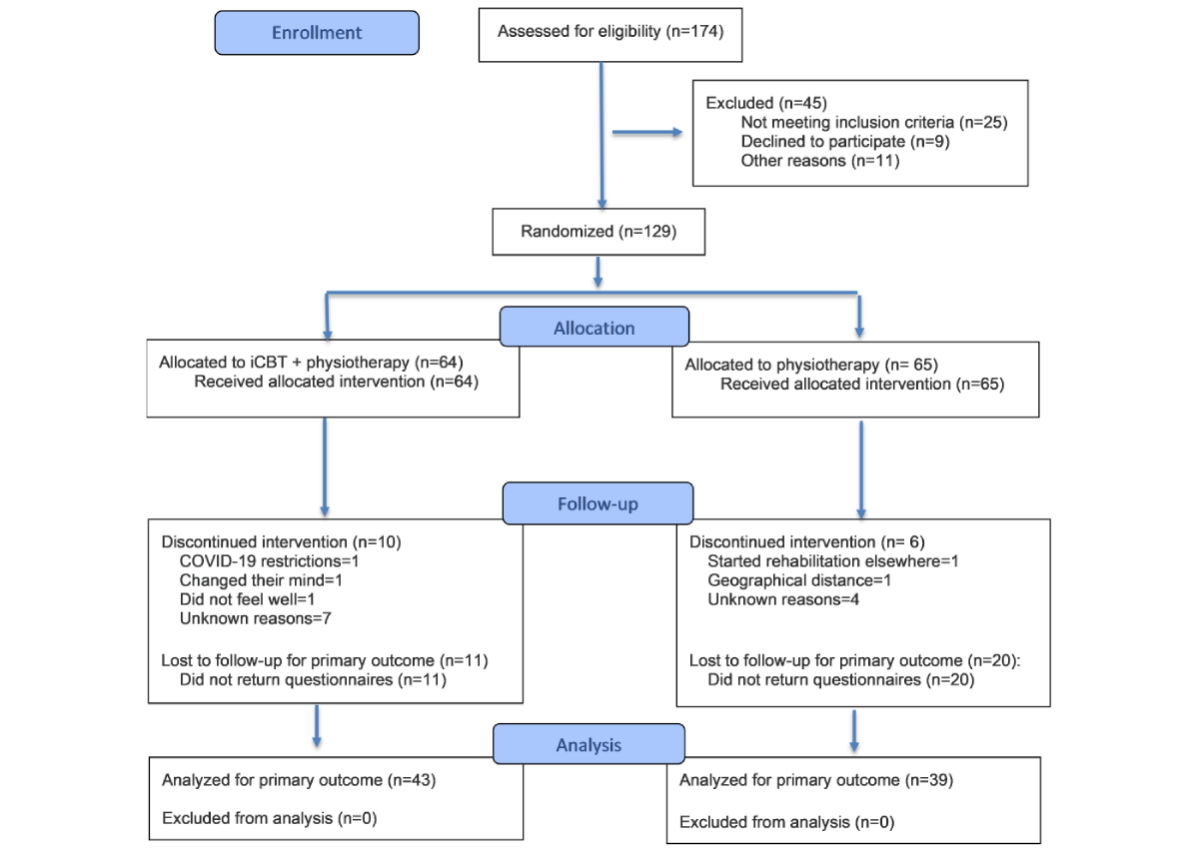
CONSORT (Consolidated Standards of Reporting Trials) 2025. Participant flow in the randomized controlled trial in Swedish primary health care, including participants with chronic widespread pain. iCBT: internet-based cognitive behavioral therapy.

A total of 9 participants who scored >14 were contacted before randomization by a psychologist by telephone for a dialogue about the severity of their psychological distress. For 1 participant, the psychologist and the participant jointly decided that inclusion in the study was not appropriate, due to the participant’s mental health. Instead, the participant was referred to a relevant service within primary health care. The other 8 participants were included in the study. The recruitment process was delayed and obstructed by the COVID-19 pandemic, resulting in an interruption before the desired sample size of 200 participants could be reached. Data collection for baseline started in November 2020, and 6 months follow-up was completed in May 2023. A total of 129 participants were included and randomized in the study. A total of 82 (64%) participants completed the 6-month follow-up and were included in the analyses; 43 in the intervention group and 39 in the control group. The participants who were lost to follow-up did not return the questionnaires and did not give reasons for dropping out. The participant flow is described in Figure 2 [23].

There were no statistically significant baseline differences in age, number of pain locations, pain duration, FIQ pain intensity, FIQ fatigue, SCI-93, level of education, work status, sick leave, or disability pension between the 82 who participated in the 6-month follow-up and the 47 who were lost to the 6-month follow-up.

Little MCAR test was not statistically significant (*χ*^2^_950_=1007.56; *P*=.10). Hence, the data could be MCAR, or the power was too low to detect a distinction from this (n=129). A statistically significant test would have indicated not MCAR.

Descriptive data of the participants are presented in Table 1. There were no statistically significant differences in descriptive data between the intervention group and the control group at baseline.

**Table 1:** Baseline descriptive data for the participants with chronic widespread pain (n=129), in the randomized controlled trial in primary health care in Sweden, presented separately for the intervention group receiving internet-based cognitive behavioral therapy (iCBT) in combination with a face-to-face physical activity plan, and the control group receiving only the physical activity plan.

		iCBT + physical activity plan (n=64)	Physical activity plan (n=65)
Age (years), mean (SD; range)		51 (10; 30-70)	51 (12; 21-69)
Pain duration (years), mean (SD; range)		14 (9; 1.5-35)^a^	12 (11; 0.3-45)^a^
Number of pain locations, mean (SD; range)		10 (4; 3-18)	10 (4; 4-18)
FIQ^b^ pain intensity (mm), mean (SD; range)		62 (18; 10-100)	63 (21; 18-100)
FIQ fatigue (mm), mean (SD; range)		69 (24; 13-100)	66 (25; 3-100)
SCI-93^c^ (score), mean (SD; range)		53 (22.2; 15-108)^a^	50 (20.1; 19-111)^a^
Female, n (%)		59 (92)	60 (92)
Education, n (%)			
	≤9 years	0 (0)	1 (2)
	10-12 years	15 (23)	15 (23)
	>12 years	49 (77)	49 (75)
Work, n (%)			
	0%	17 (27)	18 (28)
	1%-49%	3 (5)	7 (11)
	50%-79%	10 (15)	4 (6)
	80%-100%	34 (53)	36 (55)
Sick leave, n (%)			
	0%	54 (84)	57 (88)
	1%-49%	2 (3)	1 (2)
	50%-79%	3 (5)	1 (2)
	80%-100%	5 (8)	6 (9)
Disability pension, n (%)			
	0%	59 (92)	60 (92)
	1%-49%	0 (0)	1 (2)
	50%-79%	2 (3)	0 (0)
	80%-100%	3 (5)	4 (6)

^a^Data were available for 62 or 63 participants due to missing data.

^b^FIQ: Fibromyalgia Impact Questionnaire.

^c^SCI-93: Stress and Crisis Inventory-93.

### Adherence to the Intervention

After 6 months, 16 (37%) participants of the 43 in the intervention group had completed all 14 modules in the iCBT program, 19 (42%) participants had completed between 7 and 13 modules, and 8 (19%) participants had completed 0-6 modules. A total of 88 participants (46 in the intervention group and 42 in the control group) completed all 3 physiotherapy visits (baseline, 8 weeks, and 12 weeks).

### Primary Outcomes

#### Pain Intensity

No statistically significant difference between the groups was found for change in pain intensity after 6 months. Within-group improvements in pain intensity were found in both groups (Table 2). 

**Figure 3:**
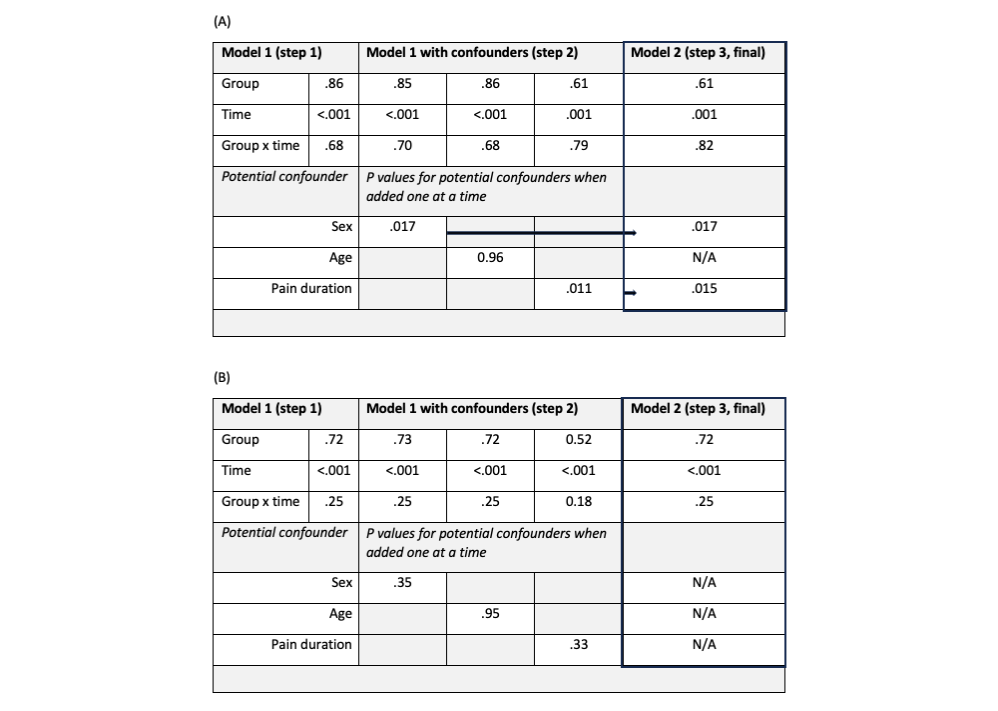
Mixed effect model analysis of (A) pain intensity and (B) number of pain locations. Baseline to 6-month follow-up in a randomized controlled trial of participants with chronic widespread pain in primary health care in Sweden. N/A: not applicable.

The mixed effects model analysis of pain intensity from baseline to 6-month follow-up is presented in Figure 3A. When potential confounders were examined individually, sex and pain duration were associated with pain intensity and were therefore included in the adjusted final model. There was a significant main effect of time, indicating a reduction in pain intensity over the study period. No statistically significant main effect of group was observed in any of the models. The group-by-time interaction was not statistically significant, suggesting that changes in pain intensity over time did not differ between the groups. A total of 9 (21%) participants in the intervention group and 13 (33%) participants in the control group (*P*=.79) improved at least 30% in pain intensity, which indicates a moderate improvement. Of these, 6 (14%) participants in the intervention group, and 7 (18%) in the control group (*P*=.89) improved at least 50% in pain intensity, which indicates a major improvement [46].

**Table 2:** Outcome values at baseline and at 6-month follow-up for the intervention group receiving internet-based cognitive behavioral therapy (iCBT) in combination with a face-to-face physical activity plan, and the control group receiving only the physical activity plan. *P* values for within-group changes and between-group differences, and Cohen d with 95% CIs for change from baseline to 6-month follow-up in a randomized controlled trial of participants with chronic widespread pain in primary health care in Sweden. Paired *t* test was used for within-group analyses and independent *t* test for between-group analyses.

	iCBT + physical activity plan (n=43)				Physical activity plan (n=39)				Between-group difference in change, mean (95% CI)		Between-group, *P* value		Cohen *d* (95% CI)
	Baseline, mean (SD)	6 months follow-up, mean (SD)	Within group, *P* value	Baseline, mean (SD)		6 months follow-up, mean (SD)	Within group, *P* value						
FIQ^a^ pain intensity (mm)	62.6 (17.7)	54.9 (23.8)	.04	62.8 (18.0)		53.5 (27.1)	.007	1.7 (–7.5 to 11.0)		.49		0.08 (–0.35 to 0.52)	
Pain locations (n)	9.6 (3.6)	7.5 (3.5)	<.001	9.6 (4.1)		8.2 (4.5)	.01	–0.8 (–2.2 to 0.6)		.90		–0.24 (–0.67 to 0.20)	
SCI-93^b^ (score)	51.4 (22.2)	46.2 (25.0)	.01^c^	47.2 (17.7)		39.4 (19.9)	<.001^d^	2.0 (–3.3 to 7.4)		.24		0.17 (–0.27 to 0.62)	
FIQ total (score)	52.6 (17.4)	50.4 (21.9)	.31	50.9 (16.0)		45.9 (21.8)	.04	2.7 (–3.7 to 9.2)		.79		0.19 (–0.25 to 0 to 62)	
FIQ Global fatigue (mm)	68.4 (25.0)	69.0 (27.3)	.85	63.2 (25.9)		61.2 (30.0)	.64	3.0 (–7.2 to 13.3)		.82		0.13 (–0.30 to 0.57)	
MFI^e^ general fatigue (score)	16.9 (2.8)	15.7 (4.3)	.004	16.2 (3.5)		15.0 (4.5)	.02	0.1 (–1.1 to 1.3)		.34		0.03 (–0.40 to 0.46)	
MFI physical fatigue (score)	15.6 (3.8)	14.8 (4.7)	.16	16.2 (3.5)		14.9 (4.3)	.02	0.4 (–1.1 to 1.9)		.46		0.12 (–0.31 to 0.56)	
MFI mental fatigue (score)	13.7 (3.7)	12.9 (3.8)	.26^c^	12.6 (4.6)		11.4 (4.5)	.04	0.4 (–1.1 to 2.0)		.34		0.13 (–0.32 to 0.57)	
MFI reduced activity (score)	13.8 (3.5)	12.6 (4.2)	.03	13.5 (3.9)		13.1 (4.1)	.33	–2.8 (–0.5 to 2.1)		.06		–0.27 (–0.70 to 0.17)	
MFI reduced motivation (score)	10.9 (3.8)	10.4 (3.9)	.45	9.6 (3.6)		9.7 (4.0)	.94	–0.5 (–2.2 to 1.2)		.45		–0.12 (–0.56 to 0.31)	
HADS^f^ depression (score)	7.3 (3.8)	7.0 (4.4)	.64	5.8 (3.4)		6.4 (3.7)	.89	–0.2 (–1.7 to 1.3)		.87		–0.05 (–0.49 to 0.38)	
HADS anxiety (score)	8.1 (4.4)	6.3 (4.7)	.004	7.3 (4.6)		5.8 (3.9)	.09	–0.8 (–2.3 to 0.7)		.36		–0.23 (–0.66 to 0.21)	
SF-36^g^ physical function (score)	66.2 (18.2)	71.2 (17.2)	.04^c^	66.8 (17.7)		67.7 (19.2)	.38^d^	2.9 (–3.7 to 9.5)		.82		0.20 (–0.25 to 0.64)	
SF-36 role physical (score)	25.0 (31.7)	30.8 (37.7)	.23	19.2 (27.2)		37.8 (41.3)	<.001	–13.1 (–28.3 to 2.0)		.88		–0.39 (–0.84 to 0.06)	
SF-36 role emotional (score)	54.4 (37.3)	59.7 (42.1)	.54^c^	55.6 (42.2)		64.1 (40.0)	.18	–4.0 (–22.8 to 14.8)		.36		–0.10 (–0.54 to 0.35)	
SF-36 energy/fatigue (score)	29.3 (20.6)	36.3 (23.8)	.004	35.3 (21.0)		38.8 (27.8)	.24	3.4 (–4.0 to 10.8)		.19		0.20 (–0.23 to 0.64)	
SF-36 emotional well-being (score)	63.0 (17.3)	64.4 (23.3)	.63	64.0 (18.6)		65.8 (19.4)	.48	–0.5 (–8.2 to 7.3)		.20		–0.03 (–0.46 to 0 to 41)	
SF-36 social functioning (score)	56.4 (25.0)	57.8 (22.8)	.63^c^	57.9 (30.3)		63.6 (23.9)	.08^d^	–4.6 (–15.0 to 5.8)		.53		–0.20 (–0.64 to 0.25)	
SF-36 pain (score)	35.7 (14.1)	42.2 (18.3)	.008	30.5 (13.4)		39.0 (21.0)	.002^d^	–3.1 (–9.8 to 3.6)		.45		–0.21 (–0.65 to 0 to 25)	
SF-36 general health (score)	44.8 (18.2)	45.1 (22.9)	.67	42.6 (20.8)		45.5 (25.0)	.26	–2.2 (–9.2 to 4.9)		.56		–0.14 (–0.57 to 0.30)	
LTPAI^h^ total (hours)	6.0 (3.3)	7.0 (4.5)	.11	7.9 (5.9)		8.0 (5.4)	.89	0.9 (–1.3 to 3.1)		.07		0.18 (–0.26 to 0.61)	

^a^FIQ: Fibromyalgia Impact Questionnaire.

^b^SCI-93: Stress and Crisis Inventory-93.

^c^Calculated for 39-42 participants due to missing data.

^d^Calculated for 36-38 participants due to missing data.

^e^MFI-20: Multidimensional Fatigue Inventory.

^f^HADS: Hospital Anxiety and Depression Scale.

^g^SF-36: Short-Form 36.

^h^LTPAI: Leisure Time Physical Activity Instrument.

#### Pain Locations

No statistically significant difference between the groups was found for the change in the number of pain locations after 6 months. Within-group improvements in the number of pain locations were found in both groups (Table 2). The mixed effects model analysis of pain locations from baseline to 6-month follow-up is presented in Figure 3B. When potential confounders were examined individually, none was associated with pain locations. There was a significant main effect of time, indicating a reduction in the number of pain locations over the study period. No statistically significant main effect of group was observed in any of the models. The group-by-time interaction was not statistically significant, suggesting that changes in the number of pain locations over time did not differ between the groups.

### Secondary Outcomes

There were no differences found between the intervention and the control groups for change in any of the secondary outcomes after 6 months. Both groups showed statistically significant within-group improvements in SCI-93, MFI general fatigue, HADS anxiety, and SF-36 pain (Table 2). There was no statistically significant difference between the groups in the participants’ ratings on the PGIC after 6 months (intervention group: median 3.0, IQR 3.0-4.0; control group: median 3.5, IQR 3.0-4.0; *P*=.59).

### Additional Outcomes

Achievement of individual main goals and intermediate goals in the physical activity plan and achievement of WHO recommendations for physical activity and strengthening exercises are presented in Table 3. The participants in the intervention group were more likely to have reached their main goals and their intermediate goals at the 12-week visit than the participants in the control group. No difference was found between the groups at the 12-week visit in achievement of the WHO recommendations of aerobic physical activity and strengthening exercise (Table 3).

**Table 3:** Achievement of individual goals and World Health Organization (WHO) recommendations for physical activity and strengthening exercise at the 12-week visit for the participants with chronic widespread pain in the randomized controlled trial in primary health care in Sweden. Data are based on the 88 participants who completed all 3 visits with the physiotherapist.

				iCBT^a^ + physiotherapy (n=46), n (%)		Physiotherapy (n=42), n (%)		Comparison between groups, *P* value
Main goals								.02
		Completely	17 (37)		8 (19)			
		Partially	23 (50)		21 (50)			
		Not at all	6 (13)		13 (31)			
Intermediate goals								.01
		Completely	25 (54)		15 (36)			
		Partially	17 (37)		13 (31)			
		Not at all	4 (9)		14 (33)			
Achieving the WHO recommendations								.67
	Yes		24 (52)		20 (48)			
	No		22 (48)		22 (52)			

^a^iCBT: internet-based cognitive behavioral therapy.

## Discussion

### Principal Findings

This study hypothesized that the combined intervention of therapist-guided iCBT plus a face-to-face physiotherapist-guided person-centered physical activity plan would result in superior improvements in pain, stress-related symptoms, psychological distress, and physical activity among individuals with CWP than the stand-alone physical activity plan. The findings did not support this hypothesis, as no significant differences in improvement were observed between the groups for either the primary outcomes of pain intensity and pain locations or the secondary outcomes of associated symptoms and amount of physical activity. However, both groups demonstrated modest improvements across several outcomes.

These findings are partly in line with a systematic review investigating the effect of CBT on patients with combined pain and stress-related disorders, in which CBT was found to have no effect on pain, but rather on psychological symptoms [20]. The improvements observed in this study are also supported by a systematic review and meta-analysis of CBT combined with exercise in adults with chronic pain, which reported small to moderate improvements in pain intensity, functional disability, and psychological outcomes such as anxiety and self-efficacy [21]. In this study, the absence of additional effects of iCBT beyond a structured physical activity intervention may suggest that physical activity alone contributes substantially to improvements in both physical and psychological outcomes, potentially limiting the detectable added benefit of iCBT in this context. However, as this study may have been underpowered, a larger sample size might have revealed a between-group difference.

There was a statistically significant difference between the groups in achievement of individual goals at the 12-week visit with the physiotherapist, favoring the combined intervention. Similar findings have been reported in a previous study showing that a CBT-based pain management program may contribute to the attainment of personally meaningful goals among individuals with chronic pain [48]. In the intervention group, the iCBT program started about 8 weeks after the participant’s first physiotherapist visit. The iCBT started later because it was considered too demanding to initiate both new physical activity habits and behavioral therapy at the same time. The participants got access to 2 new modules in the iCBT program per week. By the 12-week physiotherapist visit, the participants had access to approximately 8 modules of the total of 12 modules. Thus, the iCBT program could have contributed to improvements or behavioral changes among the participants in the intervention group that enabled them to achieve their individual goals. Approximately half of the participants in each group reached the WHO recommendations for physical activity [29] at the third visit. The physiotherapist-guided person-centered physical activity plan may have contributed to the observed improvements, aligning with previous research on patients with CWP [35]. However, it remains difficult to determine with certainty whether the improvements were attributable to the activity plan, since cohorts with chronic pain have previously shown small symptom improvements over time, particularly in studies with long-term follow-up [12,49].

### Limitations and Strengths

The main limitations of this RCT are that it was underpowered due to difficulties with participant recruitment during the COVID-19 pandemic and that the loss to follow-up was high, which affects the validity of the study results. The subgroup analyses based on the SCI-93 score were not performed since statistical power was not reached. One possible reason for the high loss to follow-up is that the questionnaires were completed on paper and had to be returned by mail, which may have required additional effort from the participants. Due to limited statistical power, the study may not have been able to detect potential differences in effects between 2 groups receiving active treatments. This was also seen in a systematic review that found iCBT to be promising in ameliorating chronic pain and its associated symptoms when compared to passive control treatments, while it may not yield superior effects when compared to active treatments [18].

The follow-up assessment was conducted 6 months after baseline. This time frame was selected because behavioral change and the establishment of regular physical activity routines were expected to require time. However, it would also have been valuable to collect additional follow-up data at 16 weeks in order to assess outcomes immediately after the completion of the intervention. Another limitation is that the questions used to determine whether participants fulfilled the WHO recommendations for physical activity were not validated. In addition, these questions were only collected at the third physiotherapy visit. Consequently, adherence to the WHO recommendations at baseline could not be determined. While the physical activity plan, including the goal setting, has been used and evaluated in terms of content and patient experiences [34,36], the specific rating of goal achievement used in this study has not previously been applied, and its validity has not yet been established, which may affect the level of confidence in the findings.

Although all participants in this RCT fulfilled the inclusion criteria for CWP, they constituted a heterogeneous population. Pain duration varied from 4 months to 45 years, and pain spread ranged from 3 to 18 locations. The variance in symptom severity across participants could have affected the outcomes, as those with more severe symptoms and longer pain duration may have required greater support than what was provided in this intervention. There was also a large range in the study sample regarding the extent of stress-related symptoms as rated on the SCI-93. This was part of the study plan, since the research question concerned whether the participants could benefit from an iCBT program for stress regardless of their stress level. The variability within this CWP population is consistent with previous findings suggesting the existence of subgroups with differing symptom severities in fibromyalgia, a subgroup of CWP [50], thereby supporting the applicability of this study’s results. A possible bias could be that the participants were aware that iCBT constituted the intervention of interest rather than the comparator intervention. However, as the intervention did not demonstrate superior effects, this potential expectancy bias may have had a limited impact on the outcomes.

Despite these limitations, this study has several strengths. The randomized controlled design reduces the risk of bias. The study was conducted in a real-world clinical setting in a patient population with complex symptoms, enhancing external validity. In addition, mostly validated outcome measures were used, and appropriate statistical methods were applied to account for repeated measures and potential confounders. The intervention in this study was designed to affect both psychological and physiological aspects of pain using iCBT for stress in combination with a person-centered plan for physical activity. After 6 months, 37% of the participants had completed all 12 modules in the iCBT program, which is in line with previous data from the same region in Sweden showing that 34% of patients participating in iCBT in primary health care completed all modules in an iCBT program [51], suggesting that adherence in this study reflects real-world implementation of iCBT in similar clinical settings. The CBT was chosen to be internet-based in this study since the participants were recruited from a large geographical area in western Sweden, involving many primary health care centers. iCBT could easily be provided to all participants regardless of location. Both the person-centered health plan and the iCBT are provided in Swedish primary health care and require minimal health care resources, offering a potentially cost-effective approach.

### Conclusions

This study contributes a novel perspective by examining whether a stress-focused intervention influences pain-related outcomes, addressing an area that has received limited attention in previous research. No differences in pain intensity, number of pain locations, or associated symptoms were observed between the intervention group receiving therapist-guided iCBT focused on stress combined with person-centered physical activity and the control group receiving physical activity alone. However, both groups demonstrated improvements over time across several outcomes. Participants in the intervention group achieved their individually set goals to a greater extent than those in the control group, suggesting a potential added value of the intervention in supporting goal attainment. In a clinical context, these findings support person-centered, collaboratively developed physical activity plans as a central component in the management of CWP, while stress-management interventions may be considered as complementary support to facilitate behavioral change and goal attainment. Although iCBT did not provide additional improvements in pain or other standard outcomes, it may still be beneficial for selected patients, particularly those with higher levels of perceived stress, which warrants further investigation. The relatively high rate of loss to follow-up warrants caution when interpreting the findings, as it may affect both the internal validity and the generalizability of the results.

## Appendix

Multimedia Appendix 1 CONSORT 2025 checklist.

Multimedia Appendix 2 CONSORT E-HEALTH checklist.

Multimedia Appendix 3 TIDieR checklist.

Multimedia Appendix 4 The 14 modules in the internet-based cognitive behavioral therapy program.

## Abbreviations

ACR: American College of Rheumatology
CBT: cognitive behavioral therapy
CONSORT: Consolidated Standards of Reporting Trials
CONSORT-EHEALTH: Consolidated Standards of Reporting Trials of Electronic and Mobile Health Applications and Online Telehealth
CWP: chronic widespread pain
FIQ: Fibromyalgia Impact Questionnaire
HADS: Hospital Anxiety and Depression Scale
iCBT: internet-based cognitive behavioral therapy
MCAR: missing completely at random
MFI: Multidimensional Fatigue Inventory
PGIC: Patient Global Impression of Change
RCT: randomized controlled trial
SCI: Stress and Crisis Inventory
SF-36: Short Form-36
TIDieR: Template for Intervention Description and Replication
VAS: visual analog scale
WHO: World Health Organization

## Data Availability

The data used in this study are not readily available, as the sharing of patient data is restricted under Swedish law and the General Data Protection Regulation. Furthermore, access to the data was restricted in the ethical approval to members of the research group only. However, deidentified data may be made available by the corresponding author upon reasonable request and subject to appropriate confidentiality agreements.
