# Supplementary figures and images for: Combined Internet-Based Cognitive Behavioral Therapy and Face-to-Face Physiotherapy in Primary Health Care for Chronic Widespread Pain: Randomized Controlled Trial
Source: J Med Internet Res. 2026 Jun 29;28:e86792. doi: 10.2196/86792 (PMC13365888; doi:10.2196/86792)

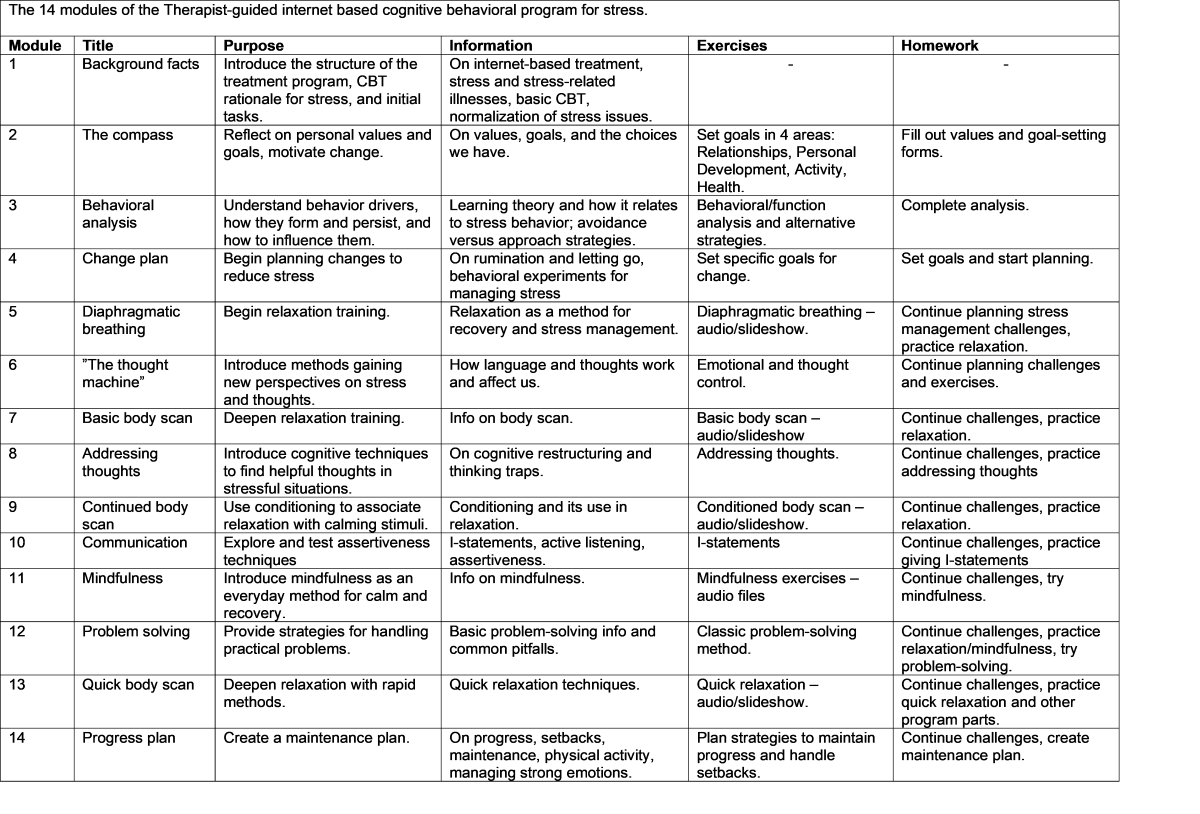

Supplement: Multimedia Appendix 4 [file jmir_v28i1e86792_app4.png]
